# Cutaneous Malignancy due to Arsenicosis in Bangladesh: 12-Year Study in Tertiary Level Hospital

**DOI:** 10.1155/2018/4678362

**Published:** 2018-12-16

**Authors:** Md. Iqbal Mahmud Choudhury, Nilufar Shabnam, Tazin Ahsan, S. M. Abu Ahsan, Md. Saiful Kabir, Rashed Md. Khan, Md. Abdal Miah, Mohd. Kamal Uddin, Md. Aminur Rashid Liton

**Affiliations:** ^1^Assistant Professor, Plastic Surgery Unit, Bangabandhu Sheikh Mujib Medical University (BSMMU), Dhaka, Bangladesh; ^2^Assistant Professor, Department of Surgery, BIRDEM Hospital and Ibrahim Medical College, Dhaka, Bangladesh; ^3^Honorary Medical Officer, Department of Surgery, Bangabandhu Sheikh Mujib Medical University (BSMMU), Dhaka, Bangladesh; ^4^Associate Professor and Head of the Department of Surgery, Ad-din Sakina Medical College, Jessore, Bangladesh; ^5^Professor and Head of Department of Dermatology and Venereology, National Medical College and Hospital, Dhaka, Bangladesh; ^6^Professor and Head of the Department of Dermatology & VD, Dhaka Medical College Hospital, Dhaka, Bangladesh; ^7^Assistant Professor, Department of Dermatology and Venereology, Centre for Medical Education, Dhaka, Bangladesh; ^8^Senior Consultant, Department of Dermatology, 250 bedded General Hospital, Gopalganj, Bangladesh; ^9^Assistant Professor, Department of Dermatology and Venereology, Holy Family Red Crescent Medical College and Hospital, Dhaka, Bangladesh

## Abstract

Bangladesh is grappling with the largest mass poisoning of a population in the world due to contamination of drinking water with naturally occurring inorganic arsenic. It is estimated that 75 million people of 59 (out of 64) districts are at risk of drinking contaminated water with arsenic above 50*μ*g/L. Long term exposure to arsenic causes cancers, including skin, lung, and bladder. This is a randomized prospective study to see the prevalence of skin cancer from arsenic affected area of Bangladesh, as well as their variation by geographical area, age, gender, location on the body, and socioeconomic conditions, in outpatient department of plastic surgery unit of Bangabandhu Sheikh Mujib Medical University (BSMMU). A total of 960 patients with skin cancers comprised of 528 males and 432 females were selected for the study from January 2004 to December 2015. In this 12-year study, we found squamous cell carcinoma, basal cell carcinoma, melanoma, and Merkel cell carcinoma to be associated with the ingestion of arsenic contaminated ground water. This is a reflection of a small part of the total national scenario of devastating result of arsenic mediated cancer in terms of skin malignancy. This study will help the future researchers who are contemplating to work on arsenic induced health problem.

## 1. Introduction

Arsenic contamination of ground water in Bangladesh is reported to be the worst problem in the world in terms of affected population [1, 2]. Survey data from 2000 to 2010 have demonstrated that an estimated 35 to 77 million Bangladeshi people, especially in rural area, have been chronically exposed to arsenic through drinking water and daily food [3–5]. Before 1970s, people in this country used surface water for drinking and daily activities. The surface water was pathogen laden and as a result they suffered from several types of diarrheal diseases in epidemic manner. To solve this problem, since 1970s, the government and other international organizations introduced shallow tube wells. The people got the apparent benefit but the hidden problem was discovered years later. The water from shallow tube wells (depth of 10-70m) contains arsenic in toxic level, which causes a lot of hazards to the human body. But ground water from deep tube wells (depths >150m) is safe and contains arsenic in less concentration [5, 6].

According to WHO guidelines, water containing arsenic over 10*μ*g/L is said to be contaminated but in Bangladesh, this bench mark rises to 50*μ*g/L [7–12]. Natural Drinking Water Quality Survey report used an estimated population of 164 million to estimate that 22 million and 5.6 million people are drinking water with arsenic concentrations >50*μ*g/L and >200*μ*g/L, respectively [13]. This can lead to massive chronic poisoning—called** arsenicosis**—causing neoplastic and nonneoplastic health problems in local population. This can occur at genetic and epigenetic level [14]. The arsenic biotransformation process which involves methylation changes is thought to play a key role for malignant transformation. Arsenic can damage DNA through oxidative stress by generation of toxic species such as reactive oxygen species (ROS) [10, 15–17]. According to the United States Environmental Protection Agency, the risk of developing skin cancer in association with daily consumption of around 1.0 liters of arsenic contaminated water at 50*μ*g/L concentration has been estimated to be 1 in 1000 to 2 in 1000[18]. Basal cell carcinoma (BCC) and squamous cell carcinoma (SCC) are the most common nonmelanotic cutaneous malignancy in patients with long term exposure to arsenic. Merkel's Cell Carcinoma (MCC), which is uncommon, and melanoma have been documented at low frequency [19–22].

In 1983, Krishna Chandra Shah identified arsenic induced skin lesions in India and by 1987, he detected several arsenic induced skin lesions in patients who came from neighboring Bangladesh. In 1993, the Public Health Engineering Department of Bangladesh officially declared the confirmation of arsenic contamination in Nawabganj district [23–25]. In 1998, British Geological Survey (BGS) collected water samples from 41 arsenic affected districts [26] and 35% of water sample were found to have arsenic contamination above 50*μ*g/L. The recent statistic on arsenic contamination indicates that 59 out of 64 districts of Bangladesh have been affected [27]. Approximately 85% area of Bangladesh has arsenic contaminated ground water and around 75 million people are at risk of arsenic ingestion from the tube-wells water [1]. Apart from the national survey there have been a number of thana and village surveys in many districts. The districts where most upazillas and villages have a large number of contaminated wells (up to 100%) are termed as** worst affected **districts. Those districts are Chandpur (90%), Munshiganj (83%), Gopalganj (79%), Brahmanbaria (70%), Jhenaidah (69%), Madaripur (69%), Noakhali (69%), Satkhira (67%), Comilla (65%), Shariatpur (65%), Lakshmipur (64%), Pabna (62%), Meherpur (60%), Bagerhat (60%), and Natore (58%). The** least affected** districts are the ones where none of the samples exceed the Bangladesh limit. They are Thakurgaon, Panchagarh, Nilphamari, Lalmonirhat, Patuakhali, Barguna, Barisal, Chuadanga, Jessore, Feni, Khulna, Magura, Narail, Rajbari, and Pirojpur [28]. In about half of the measurements, the arsenic concentration in tube-well water was about 50*μ*g/L [29, 30], which reached above 150-300*μ*g/L in some districts or parts of some districts along the border of West Bengal, India [21, 31].

The occurrence of arsenic diseases depends on the ingestion of arsenic compounds and their excretion from the body. It has been reported that 40% to 60% arsenic can be retained by the human body [32, 33]. The daily consumption of arsenic contaminated water is very high in Bangladesh, especially in villages. The villagers are more involved in manual labour and for the hard-work they consume more water (more than 5 liters per day), especially in the summer [1]. They retain arsenic not only by drinking water but by all of their food chains and cooked food which is contaminated by arsenic. We have almost no data to state exactly when the problem with arsenic poisoning started in Bangladesh, but it may be assumed that the consumption of arsenic contaminated water since 1970 is the beginning of the problem.

## 2. Materials and Methods

We performed a prospective study. Study period was from January 2004 to December 2015. The cases were selected random-wise from outpatient department (plastic surgery) of Bangabandhu Sheikh Mujib Medical University (BSMMU), Dhaka, Bangladesh. All the patients were referred from the department of dermatology of BSMMU, different medical colleges, and private chambers of dermatologists in Bangladesh with histopathology reports of incisional biopsy (**incisional biopsy** is a surgical procedure to remove a piece of tissue from a lesion or mass. The tissue is then tested by the histopathologists to find out what it is). All the lesions were larger than 2.5cm in diameter and required plastic surgical maneuver, that is, wide excision and reconstruction by skin graft or flap coverage. We then sent the excisional biopsy (**excisional biopsy** is a surgical procedure in which whole lesion or mass is removed then tested to find out what it is) specimen for histopathology after marking its different borders with different suture materials (Prolene, Vicryl, and Silk) to delineate the exact area of the body from where we have removed it. We used a container containing 10% formalin to transport the specimen to the department of histopathology. The main target of this histopathology was to see the deep surface and marginal clearance of this excised specimen. The histopathologists did immunohistochemistry (**immunohistochemistry** refers to the process of detecting the antigens in the cells of tissue section by exploiting the principle of antibodies binding specifically to the antigens in the biological tissue) for Merkel Cell Tumour and Melanoma. The histopathologists did the diagnosis of the cancer by world-wide standard methods.

Within these 12 years, 1403 patients were found with cutaneous malignancy among which 1156 cases were related to arsenic exposure. 960 cases fulfilled the selection criteria (which are listed below) and 196 patients who had multiple lesions due to arsenicosis were not included in the study.

### 2.1. Selection Criteria

- Those who have one of the dichotomous physician-diagnosed skin lesions [34],
- Melanosis, Keratosis, Hyperkeratosis, and Leucomelanosis
- (**Melanosis** is termed as a diffuse or spotted lesion characterized by dark pigmentation on the face, neck, limbs and trunk.** Keratosis** is defined as any diffuse or spotted lesion characterized by hard and roughened skin elevations observed on the hands and feet.** Hyperkeratosis** is defined as extensively thickened keratosis observed on the hands and/or feet that are easily visible from a distance.** Leucomelanosis** is defined as depigmentation characterized by black and white spots present anywhere in the body)
- History of ingestion of water from tube-wells for at least 10 years in arsenic prevalent districts marked by the government of Bangladesh
- Patients with single malignant lesion with histopathology report of incisional biopsy

Statistical analysis was performed using SPSS 16.0 software. Normally distributed continuous variables were expressed and categorical variables were expressed as numbers and percentages. Mann-Whitney U test used in comparison with the continuous variables and chi-square test was used in comparison with categorical variables. The level of statistical significance was considered as <0.05.

Ethical approval was taken from Institutional Review Board (IRB), BSMMU. The declaration of Helsinki was followed. All the patients were involved in the study after taking informed written consent. All the information was collected using a questionnaire by face to face interview.

## 3. Results

A sample of 960 patients from January 2004 to December 2015 presenting with cutaneous malignancy (single lesion) from different arsenic contaminated districts of Bangladesh. There were 528 (55%) male and 432 (45%) female. The mean age of patients was 47.8±12.76 years (male 49.1±12.11 and female 46.6±13.3). About 520 patients have family history of malignancy due to arsenicosis (Table 1).

Cancers were very less below the age of 24. Higher prevalence (28.99%) of patients was noted in 40-49 years age group which was found to be statistically significant (P <0.05). BCC was found in 563 (58.65%) cases, SCC in 384 (40%), melanoma in 11 (1.15%), and MCC in 2 (0.21%) cases. There was no statistically significant difference between male and female population. Melanomas were found in above 40 years of age and more in male (0.73%) population. Two MCC cases were found in the female population (Table 2).

In Table 3, face (10.31%) was least affected area where limbs (34.27%) were most. There was a statistically significant (P <0.05) difference between male and female patients. 270 (28.13%) lesions were found on the scalp and 262 (27.29%) were in the trunk. About 78.56% (754) patients came from low education level, whereas 3.64% (35) in high education level, which was found to be statistically significant (P <0.05). Melanomas were more among the lower educational group where MCCs were found in the educated group (Table 4).

In Tables 5(a) and 5(b), highest (n=67, 6.98%) number of patients came from Chandpur (90% affected area) and all types of cancer (BCC, SCC, Melanoma, and MCC) are found in this area. The lowest number of patient (n=11, 1.15%) came from Pirojpur and Patuakhali (P <0.05). About 66.55% (N=637) patient came from worst affected districts with 35.22% (N=338) BCC, 29.83% (N=286) SCC, 1.14% (N=11) melanoma, and 0.21% (N=2) MCC. But from the least affected areas only 33.65% (N=323) came with 23.44% (N=225) BCC and 10.21% (N=95) SCC. No melanoma or MCC was found in the least affected districts.

## 4. Discussion

Arsenic (As) is a metalloid. It is widely distributed in nature [35]. Arsenic compounds can be found in organic (when combined with carbon and hydrogen) and inorganic form (when combined with oxygen, chlorine, and sulfur among other elements) [36]. In Environment, arsenic can be found with an oxidation state +3 (Arsenite or, As [III]), or, +5 (Arsenate or As [V]), exhibiting different grades of toxicity [37]. An increased level of inorganic arsenic in drinking water is the major cause of arsenic toxicity [36, 38]. For this reason, IARC (International Agency for Research on Cancer) declared that arsenic is a human carcinogen (class I) and recommended threshold for arsenic concentration of drinking water ≤10*μ*g/L [39–41]. Smith AD et al. also showed arsenic levels as low as 2 *μ*g/L may be cancerous [42].

It is now widely believed that high arsenic levels in ground water in Bangladesh have natural geological source which may be due to abstraction of quaternary confined and semiconfined alluvial or deltaic aquifers. This is also inferred that arsenic is occurring in the alluvial sediments; the ultimate origin is perhaps in the out crops of hard rocks higher up the Ganga catchment [43]. A large number of chemical and biological reactions, namely, oxidation, reduction, adsorption, precipitation, methylation, and volatilization, participate in the cycling of the toxic element in ground water [35]. It is also manmade causes; i.e., the use of fertilizers, pesticides, insecticides, waste disposal, and arsenic treated wooden poles for power grids was blamed for the contamination [28]. That underground water system flows into shallow tube wells used by many. The water pulled to the surface from the wells is often contaminated with high levels of arsenic- causing problems in food chain [44].

Arsenic toxicity is close dependent and particularly on the amount of ingestion of arsenic compounds and their excretion from the body through urine, stool, skin, hair, nail, and breath. It has been reported that 40%-60% arsenic is retained in human body and passes slowly out through skin and nail [33]. Both arsenite and arsenate accumulate in dermis and epidermis, epidermal stem cells, have been proposed as a potential target for arsenic induced carcinogenesis [31, 45, 46]. Arsenic also has the potential to induce malignant transformation of human keratinocyte [47]. The carcinogenic mechanism in the cell includes the following: (a) biotransformation, methylation can activate the toxic and carcinogenic potential of arsenic [48, 49]; (b) arsenic induced oxidative stress, such as reactive oxygen species (ROS) leading to genomic alteration [50–53]; (C) epigenetic changes, DNA methylation, histone modification, and micro-RNAg [54, 55]. Moreover, malnutrition which is basically due to poverty of this region has a great effect on immunosuppression, causing arsenic induced malignancy [26]

In this 12 years study, we found 960 patients with cutaneous malignancy where the mean age was 47.8 years and showed male predominance. Basal Cell Carcinoma (BCC) was 58.65% (N=563) and Squamous Cell Carcinoma (SCC) was 40% (N= 384) of the study population. Ghosh SK et al. did a field study from July to August 2013, on arsenic contaminated area of West Bengal, India, where the mean age was 52.2 years, 37.5% BCC and 41.7% SCC. This result is nearly identical to our study probably due to similar geographical location, genetic constitution of the people, and mode of contamination of ground water in both the regions [56]. A similar study conducted in India supported our finding of male predominance. This could be explained by the observation that males of the rural areas of this region are more engaged in manual labour (day labourer, farmer, rickshaw puller, etc.) and consume more water per day than the females [57]. About 54.17% (N=520) patient had positive family history of cutaneous malignancy due to arsenicosis. This fact maybe attributed to the use of water (drinking, cooking, and other purposes) obtained from the same tube well by all the members of a family. Majority of the people of rural area of Bangladesh live in poverty, and they have to use only one common tube well for many families [58].

We found 1.15% (N-11) melanoma patients in our study, highest in 50-59-year age group. Melanoma is a relatively rare finding in other studies of the world. All the melanoma were located in the lower limbs, especially on sole and toe nail, found mainly in Chandpur, Gopalganj, Madaripur, and Sariatpur where the arsenic concentration is >50*μ*g/L, sometimes even up to 500*μ*g/L in some villages [29]. A study conducted in Iowa, IA, USA, suggested a potential link between elevated arsenic levels and melanoma, found mostly in patients aged >40 years. They suggested that arsenic's affinity for sulfhydryl group of keratin causes accumulation where scleroprotein is abundant, e.g., finger nails and toe nails. It is found when average arsenic concentration was 21*μ*g/L [19, 59–61].

In our series, we found 0.21% (N- 02) Merkel Cell Carcinoma (MCC) in the temporal region (sun exposed area) of two female patients hailing from Chandpur and Noakhali, where contaminated water contain high concentration of arsenic (>300 *μ*g/L). MCC is normally related to advanced age, sun exposure, and immunosuppression [62]. Ho SY et al. conducted a study in Taiwan and found three cases of MCC with history of ingestion of arsenic contaminated water more than 50 *μ*g/L (3.5 liters daily for male and 2.0 liters for female) with average duration of 20 years [63].

We found limb to be the most affected region (34.27%, N = 329), followed by scalp (28.13%, N=270). Face is the least affected area (10.31%, N=99). But nonarsenic mediated cancer especially BCC affects the face [64]. Limbs, scalp, and trunk are more affected probably due to hair, which accumulate arsenic and cause malignant changes in the area [61].

More than three-fourth patients in our study (78.56%, N=754) had education up to primary level or below, which is supported by another study in Bangladesh, where 69% patients studied up to primary level or below [34]. People of this education level suffer more from arsenic mediated cancer due to lack of knowledge, minimum perception to counseling, low financial capability for treatment, fail to take part in mitigation program, superstition, and malnutrition. They are mostly manual labourers, consuming more than 5 liters of arsenic contaminated water per day [1]. Cancer risk associated with daily consumption of 1.0 liter of water with organic arsenic (50*μ*g/L) has been estimated [18]. Malnutrition also plays a role in the pathogenesis of cancer by reducing the immunity [26].

## 5. Conclusion

Our study was aimed at presenting the prevalence of arsenic associated skin malignancy in patients coming to the plastic surgery outpatient department of BSMMU, a tertiary level hospital in Dhaka. This study shows only a small part of the national problem. Only the patients with detected cutaneous malignancy, large enough (more than 2.5cm in diameter) to require plastic surgical maneuver, were included, so a large number of patients with smaller lesions were beyond the scope of this study. The problem of arsenic exposure has started since the 1970s and a considerable amount of time has gone by since then, as a result a large number of people have become affected by arsenic associated skin malignancy. These patients only represent a portion of the total population. Many others with smaller sized lesions or precancerous lesions do not even present themselves at the proper place. The big number of skin malignancies of this small study population is very alarming and could be the tip of the ice-berg of the real problem. Future studies should be targeted at developing a nation-wide screening and management protocol in different levels of hospitals, both by the government and the nongovernment organizations. Drawing the attention of the concerned policymakers is really vital in this regard.

## Figures and Tables

**Table 1:** Demographic analysis of patients.

	Male	Female	Total
Number of Patients (%)	528(55)	432(45)	960 (100)
P value	*χ* ^2^ p = 0.22	*χ* ^2^ p = 0.09	*χ* ^2^ p = 0.13

Age (Mean and SD)	49.1 & 12.11	46.6 & 13.3	47.8 & 12.76

Age (Median)	64	69	67

Age Range	20-90	18-95	18-95

Family history of cutaneous malignancy due to arsenicosis	268 (27.92)	252 (26.25)	520 (54.17)

**Table 2:** Analysis of malignant lesions on the basis of age group and sex.

Age group	Squamous Cell Carcinoma				Basal Cell Carcinoma				Melanoma				Merkel Cell Carcinoma				Total	
	SCC				BCC								MCC					
	Male		Female		Male		Female		Male		Female		Male		Female			
	N	%	N	%	N	%	N	%	N	%	N	%	N	%	N	%	N	%
20-29	12	1.25	9	1.15	13	1.35	9	0.94	-	-	-	-	-	-	-	-	43	4.48

30-39	31	3.23	27	2.81	58	6.04	29	3.02	-	-	-	-	-	-	1	0.10	146	15.21

40-49	65	6.77	46	4.79	85	8.85	79	8.23	1	0.10	2	0.21	-	-	-	-	259	26.98

50-59	51	5.31	49	5.10	81	8.44	73	7.60	4	0.42	-	0	-	-	1	0.10	278	28.99

>60	54	5.63	40	4.17	71	7.40	65	6.77	2	0.21	2	0.21	-	-	-	-	234	23.38

Total	213	22.19	171	18.02	308	32.08	255	26.56	7	0.73	4	0.42	-	-	2	0.21	960	100

**Table 3:** Age and sex variation according to the location of lesion.

Age Group (yrs)	Face				Scalp				Limbs				Trunk			
	Male		Female		Male		Female		Male		Female		Male		Female	
	N	%	N	%	N	%	N	%	N	%	N	%	N	%	N	%
20-29	4	0.42	2	0.21	5	0.52	4	0.42	11	1.15	5	0.52	5	0.52	7	0.73

30-39	13	1.35	7	0.73	17	1.77	13	1.35	35	3.65	17	1.77	24	2.50	20	2.08

40-49	14	1.46	8	0.83	57	5.93	26	2.71	53	5.52	40	4.17	27	2.81	53	5.53

50-59	8	0.83	11	1.15	52	5.42	32	3.33	39	4.06	51	5.31	37	3.85	29	3.02

>60	11	1.15	21	2.19	41	4.27	23	2.40	48	4.99	30	3.13	27	2.81	33	3.44

Total	50	5.21	49	5.11	172	17.91	98	10.21	186	19.37	143	14.9	120	12.49	142	14.8

**Table 4:** Analysis of patients according to education level.

Patient	None-primary				Secondary-College				Graduation or, further			
	Male		Female		Male		Female		Male		Female	
	N	%	N	%	N	%	N	%	N	%	N	%
SCC	173	18.02	136	14.17	38	3.96	30	3.13	4	0.42	3	0.31

BCC	237	24.69	198	20.63	57	5.94	45	4.69	14	1.46	12	1.25

Melanoma	6	0.63	4	0.42	-	-	-	-	1	0.10	-	-

MCC	-	-	-	-	-	-	1	0.10	-	-	1	0.10

Total	416	43.34	338	35.22	95	9.90	76	7.92	19	1.98	16	1.66

**Table:** (a) History of arsenic associated malignancy in the worst affected districts

	Name and % of affected area	Basal Cell Carcinoma				Squamous Cell Carcinoma				Melanoma				Merkel Cell Carcinoma				Total	
		(BCC)				(SCC)								(MCC)					
		Male		Female		Male		Female		Male		Female		Male		Female			
		N	%	N	%	N	%	N	%	N	%	N	%	N	%	N	%	N	%
WORST AFFECTED DISTRICTS	Chandpur (90%)	20	2.08	4	0.42	23	2.4	16	1.67	1	0.1	2	0.21	-	-	1	0.1	67	6.98
	Munshiganj (83%)	12	1.25	3	0.31	8	0.83	4	0.42	-	-	-	-	-	-	-	-	27	2.81
	Gopalganj (79%)	15	1.56	7	0.73	10	1.04	12	1.25	1	0.1	-	-	-	-	-	-	45	4.69
	Brahmanbaria (70%)	13	1.35	17	1.77	11	1.15	6	0.63	-	-	-	-	-	-	-	-	47	4.90
	Jhenaidah (69%)	16	1.67	10	1.04	3	0.31	1	0.10	-	-	-	-	-	-	-	-	30	3.13
	Madaripur (69%)	12	1.25	4	0.42	23	2.4	17	1.77	3	0.31	1	0.1	-	-	-	-	60	6.25
	Noakhali (69%)	13	1.35	11	1.15	6	0.63	8	0.83	-	-	-	-	-	-	1	0.1	39	4.06
	Satkhira (67%)	8	0.83	6	0.63	5	0.52	4	0.42	-	-	-	-	-	-	-	-	23	2.40
	Comilla (65%)	17	1.77	10	1.04	13	1.35	11	1.15	-	-	-	-	-	-	-	-	51	5.31
	Faridpur (65%)	4	0.42	5	0.52	9	0.94	5	0.52	-	-	-	-	-	-	-	-	23	2.40
	Shariatpur (65%)	14	1.46	11	1.15	14	1.46	3	0.31	2	0.21	1	0.1	-	-	-	-	45	4.69
	Lakshmipur (64%)	12	1.25	13	1.35	9	0.94	6	0.63	-	-	-	-	-	-	-	-	40	4.17
	Pabna (62%)	18	1.88	7	0.73	6	0.63	7	0.73	-	-	-	-	-	-	-	-	38	3.96
	Meherpur (60%)	6	0.63	5	0.52	11	1.15	7	0.73	-	-	-	-	-	-	-	-	29	3.02
	Bagerhat (60%)	12	1.25	6	0.63	10	1.04	12	1.25	-	-	-	-	-	-	-	-	40	4.17
	Natore (58%)	14	1.46	13	1.35	6	0.63	-	-	-	-	-	-	-	-	-	-	33	3.44
	Total	206	21.46	132	13.76	167	17.42	119	12.41	7	0.72	4	0.41	-	-	2	0.2	637	66.35

**Table:** (b) History of arsenic associated malignancy in the least affected districts

		BCC				SCC				Melanoma				MCC				Total	
		Male		Female		Male		Female		Male		Female		Male		Female			
		N	%	N	%	N	%	N	%	N	%	N	%	N	%	N	%	N	%
LEAST AFFECTED DISTRICTS	Thakurgaon	6	0.63	11	1.15	3	0.31	-	-	-	-	-	-	-	-	-	-	20	2.08
	Panchagarh	5	0.52	9	0.94	4	0.42	1	0.10	-	-	-	-	-	-	-	-	19	1.98
	Nilphamari	13	1.35	6	0.63	4	0.42	8	0.83	-	-	-	-	-	-	-	-	31	3.23
	Lalmonirhat	6	0.63	10	1.04	2	0.21	4	0.42	-	-	-	-	-	-	-	-	22	2.29
	Patuakhali	7	0.73	3	0.31	1	0.10	-	-	-	-	-	-	-	-	-	-	11	1.15
	Barguna	2	0.21	9	0.94	1	0.10	4	0.42	-	-	-	-	-	-	-	-	16	1.67
	Barisal	6	0.63	10	1.04	-	-	3	0.31	-	-	-	-	-	-	-	-	19	1.98
	Chuadanga	7	0.73	9	0.94	3	0.31	1	0.10	-	-	-	-	-	-	-	-	20	2.08
	Jessore	10	1.04	9	0.94	-	-	5	0.52	-	-	-	-	-	-	-	-	24	2.50
	Feni	13	1.35	11	1.15	10	1.04	8	0.83	-	-	-	-	-	-	-	-	42	4.38
	Khulna	4	0.42	6	0.63	-	-	-	-	-	-	-	-	-	-	-	-	10	1.04
	Magura	9	0.94	13	1.35	3	0.31	2	0.21	-	-	-	-	-	-	-	-	27	2.81
	Narail	11	1.15	8	0.83	4	0.42	5	0.52	-	-	-	-	-	-	-	-	28	2.92
	Rajbari	2	0.21	6	0.63	9	0.94	6	0.63	-		-	-	-	-	-	-	23	2.40
	Pirojpur	1	0.10	3	0.31	2	0.21	5	0.52	-	-	-	-	-	-	-	-	11	1.15

	Total	102	10.64	123	12.83	46	4.79	52	5.41	-	-	-	-	-	-	-	-	323	33.65

## Data Availability

The data used to support the findings of this study are available from the corresponding author upon request.
